# The Role of PKC-θ in CD4+ T Cells and HIV Infection: To the Nucleus and Back Again

**DOI:** 10.3389/fimmu.2015.00391

**Published:** 2015-07-30

**Authors:** Chansavath Phetsouphanh, Anthony D. Kelleher

**Affiliations:** ^1^The Kirby Institute of Infectious Diseases in Society, University of New South Wales, Sydney, NSW, Australia

**Keywords:** protein kinase C, T cells, HIV-1, immunology, PKC theta

## Abstract

Protein kinase C (PKC)-θ is the only member of the PKC family that has the ability to translocate to the immunological synapse between T cells and antigen-presenting cells upon T cell receptor and MHC-II recognition. PKC-θ interacts functionally and physically with other downstream effector molecules to mediate T cell activation, differentiation, and migration. It plays a critical role in the generation of Th2 and Th17 responses and is less important in Th1 and CTL responses. PKC-θ has been recently shown to play a role in the nucleus, where it mediates inducible gene expression in the development of memory CD4+ T cells. This novel PKC (nPKC) can up-regulate HIV-1 transcription and PKC-θ activators such as Prostratin have been used in early HIV-1 reservoir eradication studies. The exact manner of the activation of virus by these compounds and the role of PKC-θ, particularly its nuclear form and its association with NF-κB in both the cytoplasmic and nuclear compartments, needs further precise elucidation especially given the very important role of NF-κB in regulating transcription from the integrated retrovirus. Continued studies of this nPKC isoform will give further insight into the complexity of T cell signaling kinases.

## Introduction

CD4+ T cells play a central role in the function of the immune system; they help B cells to produce antibodies, they can also orchestrate CD8+ T cells and macrophages against a wide variety of pathogens, and CD4+ T cells can also become cytotoxic cells that are capable of direct cell killing (1–4). However, these functional roles depend on the ability of the T cell receptor (TCR) to recognize its cognate antigen (5, 6).

T cell activation initiates when the TCR and associated CD3 proteins recognize a peptide/major histo-compatibility complex (MHC) on antigen-presenting cells (APCs) causing rapid clustering of TCR-associated molecules and associated co-receptors to form the immunological synapse (IS) (7). The T-cell component of the synapse is focused on clustering of CD3 (α, γ, δ, and ζ) and TCR (α, β), which bind specifically to the peptide/MHC complex, as well as CD4, which stabilize this interaction by binding to non-polymorphic regions of MHC class II (7, 8). This interaction triggers the TCR signaling process.

Recognition of cognate antigenic peptides on MHC molecules triggers TCR signaling, but it is co-stimulatory and co-inhibitory molecules (collectively co-signaling molecules) that direct T cell function and determine cell fate (9). The discovery of CD28 as a TCR co-stimulator provided evidence of the two-signal model of T cell activation, where TCR and co-stimulatory signaling is required for full T cell activation (10). Co-signaling receptors are now broadly defined as cell-surface molecules that have the ability to transduce signals, positively (co-stimulatory receptors) or negatively (co-inhibitory receptors) from the IS into T cells (11).

Studies from the past few decades on TCR signaling pathways have revealed and characterized many types of effector/signaling molecules that are pivotal to T cell activation pathways (12). Early studies revealed that TCR engagement led to phospholipase C-γ1 (PLC γ1)-mediated hydrolysis of membrane inositol phospholipids that resulted in the subsequent production of inositol phosphate and diacylglycerol (DAG). These lipid esters were found to increase intracellular calcium (Ca^2+^) concentrations and activate certain protein kinase C (PKC) isoforms (13). Furthermore, Ca^2+^ and phorbol ester tumor promoters [e.g., phorbol myristate acetate (PMA)] were able to mimic the TCR and co-receptor signals that lead to full T cell activation, resulting in IL-2 production and proliferation (14, 15), which are important for the elicitation of rapid T cell responses *in vitro*. Interestingly, this mode of activation essentially bypasses the cell surface ligands required for normal T cell activation.

The discovery of PKC as a lipid and Ca^2+^-dependent serine/threonine kinase that acts as a cellular receptor for tumor-promoting phorbol esters demonstrated the important role of PKC in T cell activation (16). The importance of PKC in T cell activation was substantiated by experiments that showed depletion of cellular PKC by prolonged phorbol ester treatment and PKC inhibition via pharmacological drugs, resulted in blockage of T cell activation (17). While the role of PKC isoforms in signaling cascades in the cytoplasm have been increasingly well described in a range of cell types, more recently it has been recognized that PKC-theta (θ) can also localize to the nucleus where it appears to play a distinct role. This review will focus on the importance of protein complexes that involve kinases (PKCs) in CD4+ T cells; particular emphasis will be placed on the role of PKC-θ in the IS and its newly discovered role within the nucleus.

## The Protein Kinase C Family

Protein kinase C was discovered in 1977 by Yasutomi Nishizuka and his group, when they purified a cyclic nucleotide-independent, Ca^2+^- and lipid-dependent, kinase from rat and bovine cerebellum (18, 19). The PKC family consists of 12 related isoforms which can be divided into three groups based on isoform structure and their corresponding co-factors/activators, these include: conventional (cPKCs), novel (nPKCs), and atypical (aPKCs) (20, 21). Isoforms α, β, and γ are cPKCs, which are activated by DAG, phorbol ester (PMA), and Ca^2+^, in the presence of phosphatidyserine (18, 19). nPKC consist of ε, η, δ, and θ isoforms, all of which are only activated by DAG and phorbol esters (e.g., PMA). The aPKC group isoforms are not activated by Ca^2+^, DAG, or PMA, but depend on protein–protein interactions for activation. Members of this group include isoforms ι, ζ, and μ (21, 22). An additional group, and sometimes considered as a fourth group of PKCs are the PKC-related kinases (PRKs). This group consists of three members: PRKs1–3 (23). The structure of these PRKs differs slightly from PKCs and they bind to ras-homology member A (RhoA) for activation (24, 25).

## The Novel PKC Member: PKC-θ

Protein kinase C-θ was first isolated and characterized in the early 1990s by Altman et al. (26), as they were searching for nPKC isoforms that may have a distinct role in T cell development and/or activation (12). Other investigators later cloned PKC-θ cDNA from both humans and mice from various tissues (27, 28). Chromosomal mapping identified the location of human PKC-θ gene (PRKCQ) within the short arm of chromosome 10 (10p15), which is a locus that has been previously recognized as being associated with mutations that lead to T cell lymphomas and other T cell immunodeficiencies (29, 30).

Protein kinase C-θ shares its regulatory N-terminal domain and C-terminal catalytic domain with other PKC family members, and is most highly related to nPKC-δ as the V1 domain of the two enzymes shares 49% homology (26). PKC-θ is primarily expressed in lymphoid tissues, hematopoietic cells, and muscle cells (31–33). Murine studies have revealed that there is relatively restricted pattern of expression in these compartments. Subsequent analyses using different lymphoid and myeloid cell types demonstrated that there is selective expression of PKC-θ in T cells and not B cells, neutrophils, monocytes, or macrophages. Furthermore, PKC-θ expression was found in platelets and not erythrocytes (34). Within T cells, single-positive CD4+ and CD8+ peripheral blood T cells and CD4+ CD8+ double-positive thymocytes express high levels of PKC-θ protein (35).

Protein kinase C-θ is the only member of the PKC family that has the ability to translocate to the IS between T cells and APCs upon TCR and MHC-II recognition. PKC-θ can also be activated as a result of G-protein-coupled receptor and hormone receptor signaling (36). The maturation of PKC-θ involves a sequence of phosphorylation steps, which involves PDK1 (phosphoinositide-dependent kinase 1) phosphorylating the activation-loop site of PKC-θ (37, 38). After PDK1 phosphorylation, the “hydrophobic” and “turn” motifs in the C-terminal domain of PKC-θ are exposed and further autophoshporylation stabilizes the enzyme. The “mature” PKC-θ is now primed for activation and is released into the cytosol, where it is in an inactive conformation (39). Upon receptor stimulation PKC-θ translocates to the plasma membrane, due to intracellular increases in DAG. DAG binding to the C1 domain confers high-affinity interaction between PKC-θ and the membrane (40). This leads to conformational change that allows the release of the pseudo-substrate domain from the substrate-binding site. PKC-θ is now in an “active” state and is accessible for substrate binding, phosphorylation, and activation of downstream effector molecules (36, 41). PKC-θ interacts functionally and physically with other downstream effector molecules to mediate T cell activation, differentiation, and migration (23, 42, 43).

## The Role of PKC-θ in the T Cell Synapse

Immunological synapses form between a T cell and an antigen-presenting cell, following peptide recognition via the TCR and MHC molecules. The synapse acts as the interface between the two cells and is formed by specific protein aggregation. The synapse is composed of two regions: the central core supramolecular activation cluster (cSMAC) that is surrounded by a peripheral supramolecular cluster (pSMAC) (44). Lipid rafts do not increase in the IS, but rather the rafts are reorganized preferentially in the cSMAC upon TCR/CD28 stimulation (45). The cSMAC encompasses the TCR and co-stimulatory receptor tails, whereas the pSMAC contains adhesion molecules (e.g., LFA-1) (44). PKC-θ is the only member of the PKC family that is recruited to the c-SMAC of the IS (43). PKC-θ is recruited to the cSMAC/pSMAC junction and is dependent on physical association with the cytoplasmic tail of CD28 (46, 47). More specifically, PKC-θ is located in the TCR^low^ “ring” of the cSMAC where it co-localizes with CD28 for signaling initiation. The TCR^high^ compartment of the cSMAC is where signal termination and internalized signal complex degradation occur (48, 49).

Protein kinase C-θ translocation to the cSMAC is mediated by its regulatory V3 domain (proximal to the catalytic domain) and requires Lck (lymphocyte-specific protein tyrosine kinase). In stimulated T cells, Lck is recruited to the tyrosine-phosphorylated P^190^Y*AP (PR motif) found in the cytoplasmic tail of CD28, via its SH2 domain. The interaction between the PXXP motif of PKC-θ V3 and the SH3 domain of Lck is necessary for PKC-θ and CD28 co-localization (50, 51). The CD28–Lck–PKC-θ interaction is thought to be responsible for the reorganization of lipid rafts, auto-phosphorylation and activation of Lck, stabilization of IL-2 mRNA expression, and other additional biological functions (47, 49, 52).

## PKC-θ is Critical for T Cell Activation and IL-2 Production

T cell receptor engagement and CD28 interaction initiates a series of PKC-θ-dependent events that eventuate in activation of transcription factors, such as NF-κB, AP-1, and NFAT, which are critical for CD4+ T cell activation, proliferation, and differentiation (53–55). NF-κB (nuclear factor kappa b) is a major target of activated PKC-θ and this interaction leads to IL-2 production. NF-κB is generally in an inactive form within the cytoplasm, where its nuclear localization signal (NLS) is masked by the multi-subunit inhibitor κB (IκB). These inhibitors undergo degradation when IκB kinases (IKK) become activated via PKC-θ phosphorylation, leading to nuclear translocation of NF-κB (56, 57). However, this process requires other effector molecules that link via PKC-θ to NF-κB IKK complex. Phosphorylation of caspase activation and recruitment domain (CARD) and membrane-associated guanylate kinase (MAGUK) domain-containing protein-1 (CARMA1) by activated PKC-θ (58–60) promotes CARMA1 to associate with Bcl10 (B-lymphocyte lymphoma/leukemia 10) and mucosa-associated lymphoid tissue 1 (MALT1) proteins, which leads to activation of the IKK complex (58, 59).

Other transcription factors regulated by PKC-θ are AP-1 and NFAT. AP-1 (activator protein-1) is a dimer of Jun (c-Jun, JunB, and JunD) and/or Fos (c-Fos, FosB, Fra-1, Fra-2, and FosB2) family proteins. *De novo* synthesis, phosphorylation, and dephosphorylation of Jun and Fos proteins regulate AP-1 activity. There are two AP-1 binding sites located in the IL-2 enhancer region [−150 bp (proximal) and −180 bp (distal)] (61, 62). It was discovered that PKC-θ was the only PKC isoform that can regulate AP-1 enhancer activation in a Ras-dependent manner (53). NFAT (nuclear factor of activated T cells) is another transcription factor that is regulated by PKC-θ. The transactivation and nuclear translocation of NFATp and NFATc is impaired in PKC-θ-deficient mice (63). Later experiments showed that upon TCR stimulation, NFAT is dephosphorylated by calcineurin, which forms a functional partnership with PKC-θ. The association of Ca^2+^/calcineurin and PKC-θ leads to the activation and nuclear translocation of NFAT that eventuates into IL-2 production in activated T cells (64).

## Multiple Functions of PKC-θ in CD4+ T Cell Subsets

*In vitro* and *in vivo* studies using PKC-θ-deficient (*Prkc1^−^*/*^−^*) mice demonstrated that PKC-θ has differential functions in distinct T cell subsets (47, 65). PKC-θ was found to be pivotal for Th2-type immune responses to allergens and helminth infections. However, PKC-θ was dispensable for Th1-dependent resistance to *Leishmania major* and for CTL-mediated antiviral responses. PKC-θ was essential for the induction of Th17-mediated EAE (experimental autoimmune encephalitis), a model of multiple sclerosis (66, 67). *In vitro* proliferation studies demonstrated that PKC-θ was crucial for Th2 and Th17 development, but only moderately affected Th1 differentiation, as PKC-θ-deficient T cells were still able to differentiate into Th1 cells (65, 68, 69). Overall, PKC-θ plays a stronger role in the generation of Th2 and Th17 responses and is less important in Th1 and CTL responses. These observations showed the importance of PKC-θ in the regulation of the adaptive immune response in particular effector T cells, prompted the question regarding its role in regulatory T cells (Treg).

In contrast to effector T cells, PKC-θ was found to mediate negative feedback on Treg suppressive function. Activation of Tregs caused the sequestration of PKC-θ away from the cSMAC, and repression of PKC-θ via inhibitors increased the suppressive activity of Tregs (70). However, *in vivo* studies showed that PKC-θ was necessary for natural Treg development in the thymus, as PKC-θ knockout mice showed impaired Treg numbers in the periphery, although the function of the activated mature Treg in the periphery remained unaltered (43, 47, 70, 71). It appears that PKC-θ plays a negative role in Treg function; sequestration of PKC-θ improves their suppressive activity in the presence of inflammatory cytokines. Higher levels of PKC-θ were observed in Tregs compared to T effectors, however, even with CD28 co-stimulation, PKC-θ appears to be sequestered away from the IS in Tregs. There was also reduced CARMA1 recruitment in association with PKC-θ sequestration (70, 72). However, a possible reason for improved suppressive activity of Tregs is that inhibition of PKC-θ prevents Tregs from TNF-α-mediated inactivation (73). It was also observed that PKC-θ inhibition enhanced survival of mice from inflammatory colitis and restored Treg activity in rheumatoid arthritis patients (70).

## PKC-θ and T Cell Anergy

Protein kinase C-θ has also been implicated in playing a role in T cell anergy. T cell anergy is a major mechanism that maintains peripheral T cell tolerance. T cell anergy is induced by TCR engagement with inappropriate co-stimulatory signaling, rendering the T cell into an unresponsive state (74). T cell anergy is also associated with deficient IL-2 production, as the CD28/B7 signaling pathway is defective (75). As PKC-θ acts as an important effector molecule downstream of CD28 signaling, any defects in co-stimulation may inhibit PKC-θ effector functions. PKC-θ-deficient T cells are not able to up-regulate anti-apoptotic factors such as Bcl-2 and Bcl-xl and undergo accelerated apoptosis (76, 77). PKC-θ has been shown to phosphorylate the pro-apoptotic molecule BAD (Bcl-2-associated death promoter), inactivating it and thus promoting T cell survival (78). The pro-oncoprotein, c-Cbl, is another molecule that has been shown to negatively regulate T cell function. c-Cbl can associate with the adapter protein LAT (linker for activation of T cells) and can act as a negative regulator of PTKs (protein tyrosine kinases) Fyn, Syk, and Zap70 and thus cause T cell anergy (79). PKC-θ-mediated phosphorylation of serine and tyrosine residues of c-Cbl prevents its inhibitory effect. Phosphorylation of c-Cbl by PKC-θ inhibits the recruitment of Sh2-containing proteins and subsequent association of cbl E3 ubiquitin ligase with its target proteins (80).

## PKC-θ in the Nucleus

**Figure 1:**
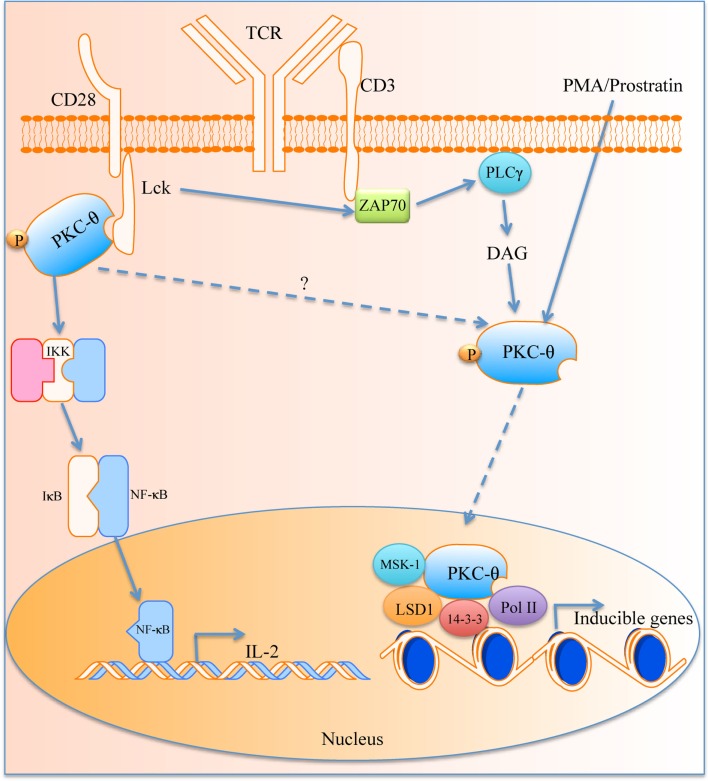
**The role of PKC-θ in T cell activation**. PKC-θ becomes activated in the immunological synapse that starts a signaling cascade, which leads to translocation of NF-κB into the nucleus for IL-2 expression and T activation. PKC-θ can also traverse the nuclear membrane and causes transcription of inducible genes via its binding partners.

Recently Sutcliffe et al. have demonstrated a new role for PKC-θ within the nucleus. PKC-θ was found to contain a NLS domain and *in silico* analyses have revealed that AKT1, HABP4, CHD3, and TCLA1 are candidate proteins that may facilitate the transport of PKC-θ into the nucleus (81). PKC-θ was associated with mediating inducible gene expression in the development of memory CD4+ T cells (82). It was found that chromatin-tethered PKC-θ forms an active nuclear complex by interacting with other nuclear proteins, including RNA polymerase II, the histone kinase MSK-1, the demethylase LSD-1, and the adaptor molecule 14-3-3ζ at regulatory regions of inducible genes, including IL-2 (Figure 1). Moreover, genome-wide analysis identified numerous nPKC-θ target genes and microRNAs that are implicated in T cell development, proliferation, differentiation, and apoptosis (81–83). The ability of PKC-θ to induce transcription was confirmed in mesenchymal transition and breast cancer stem cells, where PKC-θ was able to elicit inducible transcription programs that drive mesenchymal differentiation and cancer stem cell formation (84). However, the exact role of PKC-θ in the nucleus whether it retains its kinase activity and the existence of other potential binding partners remains to be investigated.

## PKC-θ in HIV Infection

HIV-1 negative element *Nef* gene plays a key role in disease progression. Nef has been associated with PKCs that phosphorylate Lck and the N-terminus of Nef (85). PKC-θ has been implicated as a possible physiological cofactor of Nef, and can promote NFAT-dependent gene expression and subsequently T cell activation (86). Recently, investigations using specific inhibitors demonstrated that PKC-θ has the ability to increase replication of latent HIV-1 through modulation of transcription from the integrated genome. HIV-1 also induced higher PKC-θ phosphorylation levels in infected CD4+ T cells. Inhibition of PKC-θ also hindered its translocation to the plasma membrane, which leads to a reduction in HIV-1 retro-transcription via partial repression of SAMHD1 (SAM domain and HD domain-contain protein1). Inhibitor treatment did not completely abolish T cell function as the CD4+ T cells were still able to proliferate and IFN-γ production by CD8+ T cells remained unaffected, thus avoiding total immunosuppression (87).

Phorbol myristate acetate, as well as the non-tumor-promoting deoxyphorbol ester Prostratin, an activator of PKC, and related compounds such as byrostatin and other engineered synthetic derivatives (88, 89) have been used *in vitro* to activate latently infected cells for HIV-1 eradication. These esters were shown to stimulate rapid nuclear translocation of NF-κB and activation of HIV-1 long terminal repeat (LTR) in a κB enhancer-dependent manner, via PKC. These compounds have been assessed with regard to their binding affinity to PKC-δ, rather than PKC-θ (89, 90). Therefore, the exact manner of the activation of virus by these compounds and the role of PKC-θ, particularly its nuclear form and its association with NF-κB in both the cytoplasmic and nuclear compartments, need further precise elucidation especially given the very important role of NF-κB in regulating transcription from the integrated retrovirus.

In addition, the issue of systemic activation of non-infected cells by these compounds may be advantageous or problematic. While ideally the specific cell type harboring HIV-1 should be targeted to allow specific activation of only infected cells, there may be potential advantages of more generalized T cell activation by these compounds (91, 92). As explained previously, PKC-θ appears to play a complex role in the regulation T cell subsets including the balance between Th1 and Th2 cells and in the number and function of CTL and Treg. Depending on the net effects of specific PKC-θ activators, their effects may help or hinder the clearance of cells carrying reactivated virus (93). While *in vitro* experiments may give some insight to outcomes, it is likely that *in vivo* experiments in models, such as humanized mice, will be required to dissect these effects. These findings demonstrate that PKC-θ may play an important role in HIV-1 pathogenesis, and its role in the nucleus in the context modulating HIV transcription requires further investigation.

## Conclusion

Protein kinase C-θ is a serine/threonine-specific kinase that has been extensively studied as a core component of the IS, and has been shown to play a central role in T cell activation downstream of TCR and co-stimulatory molecule engagement (76, 94). Other interesting facets of PKC-θ are its recently described function in the nucleus. The relative roles of these two facets of PKC-θ and the precise mechanism of action of this molecule in the nucleus are still being dissected. The relatively restricted distribution of PKC-θ is a potential advantage as a target for pharmacological intervention. Drugs that target PKC-θ have a potential role in modulating not only T cell function but also in the interaction of these cells with viral pathogens, particularly lymphotrophic viruses such as HIV. In addition, given the complex interactions involved in the processes regulating host and HIV transcription, targets such as PKC-θ, particularly after its translocation to the nucleus may have advantages in the manipulation of the latent integrated provirus and provide more potent and specific tools in the quest for viral eradication than the other classes of drugs currently being explored for this purpose.

## Conflict of Interest Statement

The authors declare that the research was conducted in the absence of any commercial or financial relationships that could be construed as a potential conflict of interest.
